# Spatiotemporal Variation and Hot Spot Detection of Visceral Leishmaniasis Disease in Kashi Prefecture, China

**DOI:** 10.3390/ijerph15122784

**Published:** 2018-12-08

**Authors:** Canjun Zheng, Jingying Fu, Zeng Li, Gang Lin, Dong Jiang, Xiao-nong Zhou

**Affiliations:** 1 Chinese Center for Disease Control and Prevention (China CDC), Beijing 102206, China; zhengcj@chinacdc.cn; 2 State Key Laboratory of Resources and Environmental Information System, Institute of Geographical Sciences and Natural Resources Research, Chinese Academy of Sciences, Beijing 100101, China; fujy@igsnrr.ac.cn (J.F.); jiangd@igsnrr.ac.cn (D.J.); 3 College of Resources and Environment, University of Chinese Academy of Sciences, Beijing 100049, China; 4 College of Geoscience and Surveying Engineering, China University of Mining & Technology (Beijing), Beijing 100083, China; lizeng_cumtb@163.com; 5 National Institute for Parasitic Diseases, Chinese Center for Disease Control and Prevention (China CDC), Shanghai 200025, China; zhouxn1@chinacdc.cn

**Keywords:** GIS, hot spots, spatiotemporal clusters, Kashi Prefecture

## Abstract

Visceral leishmaniasis (VL) remains a serious public health problem in China. To explore the temporal, spatial, and spatiotemporal characteristics of visceral leishmaniasis (VL), the spatial and spatiotemporal clustering distribution and their relationships with the surrounding geographic environmental factors were analyzed. In this study, the average nearest-neighbor distance (ANN), Ripley’s K-function and Moran’s I statistics were used to evaluate spatial autocorrelation in the VL distribution of the existing case patterns. Getis–Ord Gi* was used to identify the hot-spot and cold-spot areas based on Geographic Information System (GIS), and spatiotemporal retrospective permutation scan statistics was used to detect the spatiotemporal clusters. The results indicated that VL continues to be a serious public health problem in Kashi Prefecture, China, particularly in the north-central region of Jiashi County, which is a relatively high-risk area in which hot spots are distributed. Autumn and winter months were the outbreak season for VL cases. The detection of spatial and spatiotemporal patterns can provide epidemiologists and local governments with significant information for prevention measures and control strategies.

## 1. Introduction

Visceral leishmaniasis (VL) remains a serious public health problem in China. Two epidemiological types of VL are classified in western China based on the ecosystem and source of infection [1,2]. The first one is a zoonotic type caused by *Leishmania infantum* and classified into two subtypes, namely, a mountainous subtype and a desert subtype. The mountainous subtype is primarily distributed in Gansu and Sichuan Provinces, whereas the desert subtype is endemic in Xinjiang, western Inner Mongolia, and northern Gansu in the north-western desert regions of China [2]. The second type is an anthroponosis that is endemic in the oases of the plains of Kashi Prefecture, Xinjiang Uygur Autonomous Region. Cases most frequently occur in young people under 20 years of age [3]. The transmission cycle is from human to human, and no animal host has been found (0–0.3%) [4].

Kashi Prefecture, located in the southwest of the Xinjiang Uygur Autonomous Region, has been one of the most serious endemic areas for VL in China within the last century [5,6]. Compared with other areas of China in which the prevalence of VL is basically controlled, the prevalence in Kashi has not decreased but, rather, has increased in the last 10 years [7], which may have been caused by the lack of drugs and insufficient medical expenditures, global warming and agricultural population movement, environmental change, insufficient attention to the damage caused by VL, and patients with VL who cannot be treated in an appropriate time and who have become a source of infection since the 1990s [2,5,8]. For a long period, some epidemiologic studies have been focused on the temporal distribution or the purely spatial distribution of VL by means of the retrospective review and the purely spatial distribution model [5,6], but little is known about the exploration of the spatiotemporal distribution in this region. Therefore, in an epidemiological sense, it is very necessary to analyze the spatial and spatiotemporal distributions via advanced technologies such as Geographic Information System (GIS), remote sensing, and scan statistics for formulating prevention and control measures.

In this study, we conducted a GIS analysis (a powerful and efficient tool to investigate spatial patterns that include cluster, random, or dispersion-type distributions) and scan statistics of the VL outbreak by focusing on its the temporal and spatiotemporal characteristics, especially its clustering features, and also discussed their relationships with the surrounding geographic environmental factors. This work enhances the knowledge of the current status of the epidemic and contributes to a greater understanding of the areas where the outbreak occurred. 

## 2. Materials and Methods

### 2.1. Study Area

**Figure 1:**
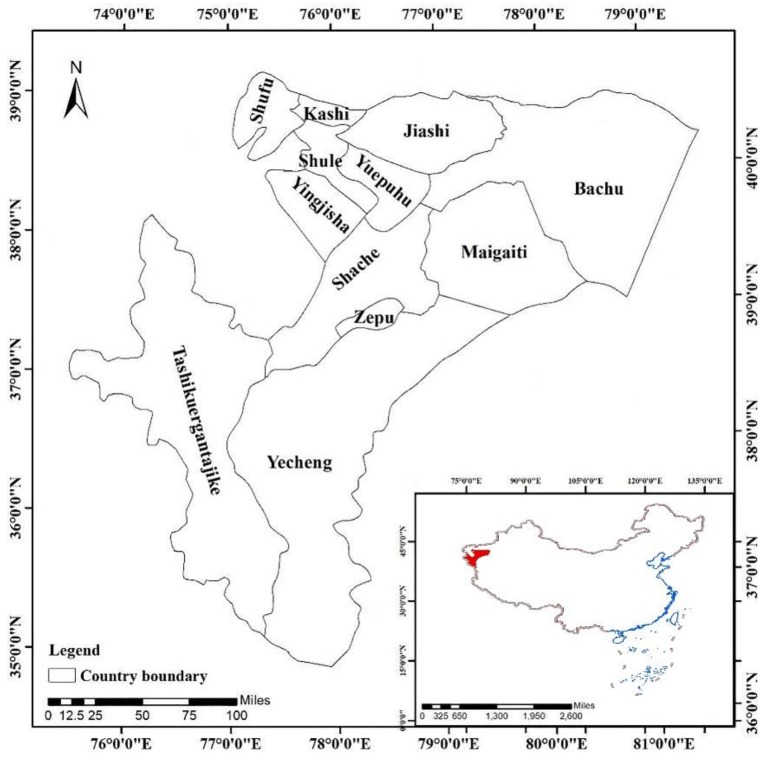
Study region, Kashi Prefecture and its counties.

Kashi Prefecture is located in the northwest of China, in the southwest of the Xinjiang Uygur Autonomous Region. For Kashi, the Taklamakan Desert is to the east, the Tianshan Mountains are to the north, the Pamirs Plateau is to the west, and the Karakoram Mountains are to the south. The prefecture is within 35°28′–40°16′ N, 71°39′–79°52′ E, with the width from east to west approximately 780 km, and the length from north to south approximately 535 km, covering an area of 162,000 km^2^ and with more than 4,488,200 people in 12 cities or counties, as shown in Figure 1. According to climatic information, the area has a warm temperature, continental, dry early climate, and the annual average temperature is approximately 11 °C. The Kashi region is currently one of the primary epidemic areas of visceral leishmaniasis in China. Several outbreaks have occurred in recent years, and the number of cases accounts for more than 90% of the total number in Xinjiang. 

### 2.2. Data Acquisition

A retrospective review of VL cases with data reported from 2005 to 2017, the latest available data, was used in this study. The data were obtained from the passive surveillance data reported through the web-based National Diseases Reporting Information System (NDRIS) operated by the Chinese Center for Disease Control and Prevention. In China, VL cases are compulsorily reported through the NDRIS according to the National Regulation on the Control of Communicable Diseases from 2005 [9]. Each record included information such as name, age, and gender of the patient, diagnosis, date of birth, date of onset, and current address code, among other factors. Geographic information data of the county were collected from Xinjiang Bureau of Surveying Mapping and Geoinformation (data source: http://www.xjmaps.com.cn/map/index.html). For conducting a GIS-based analysis of the spatial distribution of VL, the county line and polygon layer were generated on the basis of an administrative boundary map (Scale-1:25,000) obtained from the Land Revenue Office. All VL cases were geocoded using latitude–longitude coordinates in different periods of time and matched to the county-level layers of the polygon by administrative code using the software ArcGIS 10.2 (Environmental Systems Research Institute, Inc., Redlands, CA, USA).

The environmental factors GIS data used in this study mainly included the cumulative annual average raster data of the Normalized Difference Vegetation Index, temperature, precipitation, and relative humidity which were respectively obtained from National Oceanic and Atmospheric Administration (NOAA) (data source: https://www.ncdc.noaa.gov/cdr/terrestrial/normalized-difference-vegetation-index), the Resources and Environmental Scientific Data Center (RESDC), the Chinese Academy of Sciences, and the China Meteorological Administration (CMA).

### 2.3. Methodology

In this paper, the spatiotemporal clustering characteristics of VL cases were explored using the following steps:

Step 1: Descriptive statistics were used to obtain the general epidemiological characterization and the yearly and monthly temporal distributions of the disease from the study area.

Step 2: Locations of cases at the level of latitude and longitude were geocoded via GIS, and global clustering techniques were applied such as Moran’s I, average nearest-neighbor distance, and Ripley’s K-function to analyze and evaluate the distribution of the existing cases. 

Step 3: The Getis–Ord Gi* statistic was used to analyze local spatial clusters and distinguish between the locations of hot spots and cold spots.

Step 4: Kulldroff’s spatiotemporal retrospective permutation scan statistic was used to detect spatiotemporal clusters of VL. 

Step 5: The relationships between VL spatial cluster distribution and environmental factors in hot spots and spatiotemporal aggregation areas were discussed by a simple quantitative statistical analysis.

**Figure 2:**
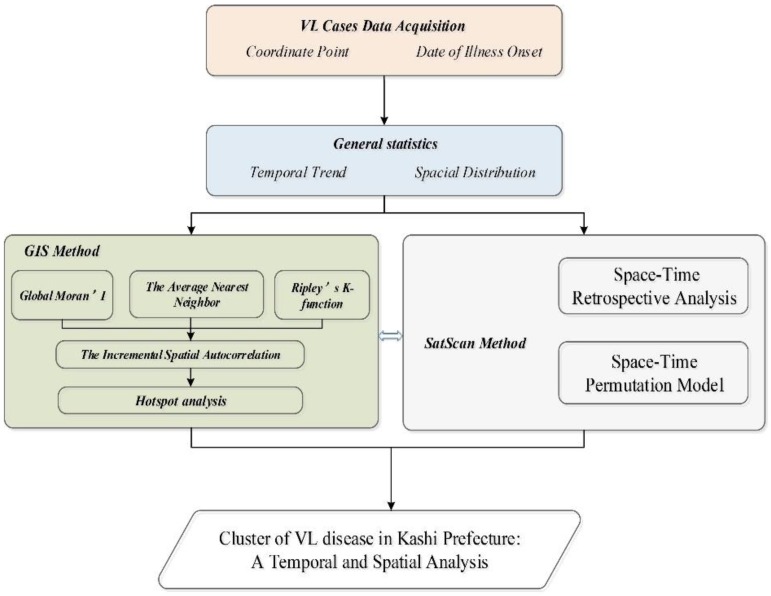
Research Technology Roadmap.

For specific research methods, please refer to the attachment; the specific process is shown in Figure 2.

## 3. Results and Analyses

### 3.1. General Status

This research was conducted on 1706 VL cases that occurred from 2005 to 2017 in Kashi Prefecture. The results showed that scattered children accounted for 55.88% of the total number of cases and that the under-10-years age group was a high-risk group that accounted for 80.13% of the total cases. Among them, the majority of cases were in infants and young children under two years of age, accounting for 53.09% of the total number of cases. The reason for this result was most likely that adults were less susceptible because of more complete immunity than infants. In the reported cases, 974 were males, accounting for 57.09% of the total number of cases, and 732 cases were females, accounting for 42.91%; thus, males were more likely to be infected than females, with a male to female ratio of 1.36:1.

### 3.2. Time Trend

**Figure 3:**
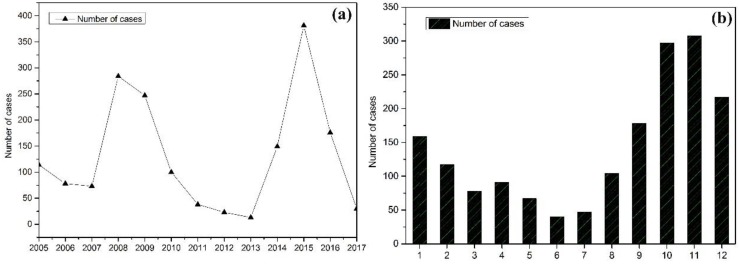
(**a**) Yearly distribution of visceral leishmaniasis (VL) cases from 2005 to 2017 and (**b**) monthly aggregated VL cases incidence.

Figure 3a shows the yearly case numbers of VL from 2005 to 2017. In general, there was an initial peak of more than 284 cases in 2008, followed by a sharp decrease to 13 cases in 2013. A second peak was then observed during a new outbreak in 2015, capping out at 381 cases in the year. After this, the cases dropped sharply in the following years. As shown in Figure 3b, we calculated the total number of cases by the month during the examined 13 years. Overall, the case numbers changed with the month: outbreaks occurred in lower temperature months, such as in autumn and winter, with the highest case numbers appearing in November, while the lowest numbers of cases occurred during the summer months.

### 3.3. Spatial Distribution

**Figure 4:**
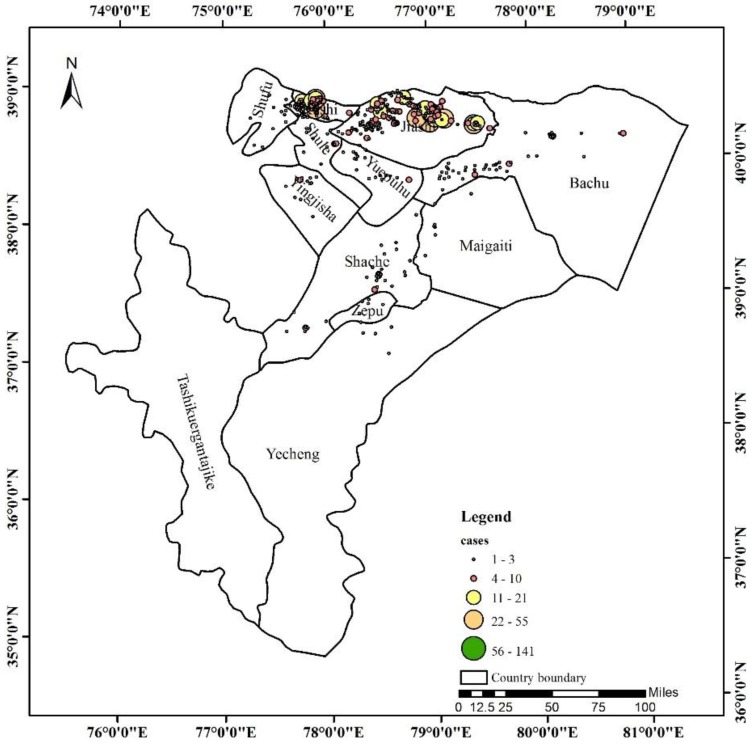
Geographical distribution of all VL cases between 2005 and 2017.

From the spatial geography analysis, the incidence of the disease was mostly concentrated in the northwest of the Kashi region (Figure 4). VL occurred more frequently in Jiashi, Kashi, and Shufu, three county-level cities, than in other areas, and the other areas were scattered and had lower incidences. Most of the cases were observed in Jiashi County (62.90% (1073/1706)), followed by Kashi City (15.65% (267/1706)) and Shufu County (8.56% (146/1706)). Figure S1 shows the geographical distribution of VL cases from (a) 2005 to (m) 2017. In general, from the space point of views, there were more VL cases in Kashi (42.5/100,000 population) each year due to the high population density and fluidity of this area, and the new cases were mainly concentrated in Jiashi (280.9/100,000 population) in the years 2008, 2009, 2010, 2014, 2015, and 2016.

For Moran’s I statistic in this study, Moran’s I value was 0.1258, with the *z*-score of 16.36, which indicated a high level of clustering (*p* < 0.0001). Therefore, this method rejected the null hypothesis and recognized that the distribution of VL cases throughout the region was clustered. For ANN, the nearest-neighbor observed mean distance was 439.68 m, the expected mean distance was 3234.18 m, the nearest-neighbor ratio was 0.1359, the *z*-score was −68.0943, and the *p*-value was 0. The ratio was less than 1, which indicated a less than 1% likelihood that this clustered pattern could be the result of random chance; thus, the spatial distribution of VL cases was aggregated. The results for Ripley’s K-function for the years 2005–2017 illustrated that the distributions were clustered at distances of 10,000–80,000 m (*p* < 0.01).

### 3.4. Hot-Spot Analysis

**Figure 5:**
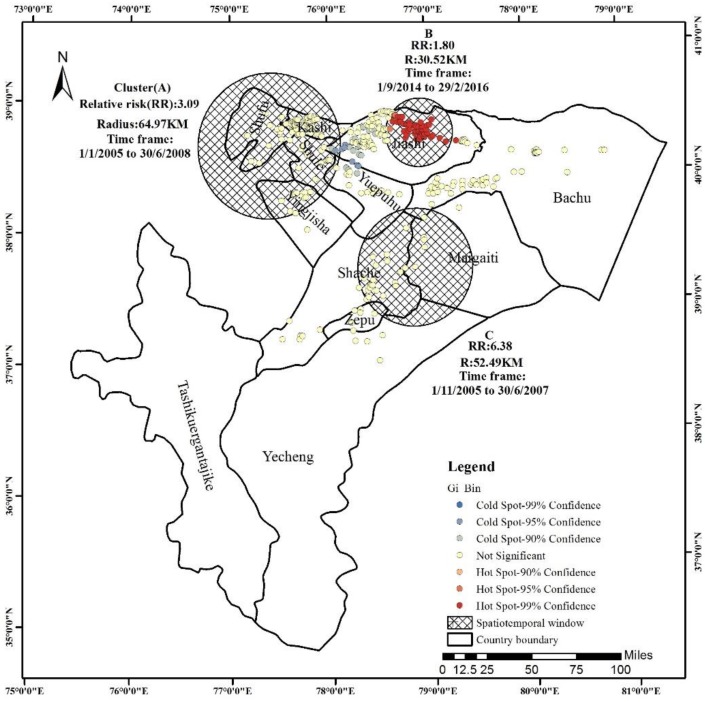
Hot spots and spatiotemporal scan statistical analysis.

The Incremental Spatial Autocorrelation tool was used to find a distance band that reflected the maximum spatial autocorrelation and noted where the resulting *z*-scores apparently peaked, and we used the distance associated with the peak value for the analysis of hot spots. The results showed that the *z*-score maximum value with the maximum peak was 9.91303 for the distance of 27,460.48 m, and this value (27,460.48 m) was selected as the parameter for the distance band in hot-spot analysis. The Getis–Ord Gi* statistic used in local spatial analysis showed outstanding spatial aggregation of VL cases in classed clusters forming “hot spots” and “cold spots”. The result of the hot-spot analysis constitutes a new finding, which indicated if the VL cases were distributed in statistically significant hot spots or cold spots or did not show statistical aggregation. In this analysis, the scale shows the confidence level bin (Gi-_Bin, which identifies statistically significant hot and cold spots regardless of whether or not the False Discovery Rate (FDR) correction is applied). Confidence values: the red dots represent hot spots, and at higher confidence values, the tendency to spatial aggregation is greater (hot spot). The blue dots represent cold spots, and at lower confidence values, the tendency to intense is greater than that to spatial aggregation (cold spot). Random distribution is shown in yellow. As shown in the map in Figure 5, significant spatial patterns of VL cases were detected that were mostly spread in the central part of the county, and the hot spots and cold spots were mostly concentrated in Jiashi County. The hot spots were in the northeast of Jiashi County and accounted for most of the total number of VL cases, and the cold spots were in the southwest of Jiashi County, with a few in the northwest of Yuepuhu County. The hot spots were areas that represented a high risk of disease. Therefore, in the future prevention and control of VL, Jiashi County is an outstanding location that requires our further attention.

### 3.5. Spatiotemporal Analysis

The distribution of VL cases in relation to village location was analyzed by the Scan statistic. The results of the spatiotemporal retrospective permutation scan statistical analysis included the most likely cluster (A) and two secondary clusters (B, C) (*p* < 0.001). Figure 5 also displays the location and size of the spatiotemporal clusters of high incidence rate for VL cases, and the parameters shown indicate the cluster name (A, B, C), radius (R), relative risk (RR), and the time frame in each cluster. 

The most likely cluster (A) located in the north-west of the region included part of Kashi City, Shufu County, and Shule County within a radius of 64.97 km and contained 223 cases that occurred from 1 January 2005 to 30 June 2008. The secondary cluster (B) located in the north of the region included part of Jiashi County within a radius of 30.52 km and contained 412 cases that occurred from 1 September 2014 to 29 February 2016. The secondary cluster (C) located in the central north of the region, included part of Shache County, Maigaiti County, Yecheng County, and Zepuhu County within a radius of 52.49 km and contained 22 cases that occurred from 1 November 2005 to 30 June 2007. 

Finally, the combination analysis of the hot-spot areas and spatiotemporal distribution was conducted using ArcGIS 10.2 (Figure 5).

The cluster B basically included the hot-spot areas, with their geographic area more consistently located in the northern part of Jiashi County, and the distance band of the maximum *z*-score in the incremental spatial autocorrelation analysis very close to the radius of Zone B. Jiashi County was the hot spot and had the highest number of observed and expected cases compared with the other clusters; thus, monitoring and control of the VL epidemic should be more focused on this region.

## 4. Discussion

Although both genders are susceptible to the disease, the total incidence of VL in males was higher than in females. The higher incidence of the disease in men might be primarily related to their behavioral characteristics; for example, regarding the living and sleeping habits of local residents, men use less covering than females in summer and therefore have increased exposure to infected phlebotomine sand flies [10]. In addition, sex-linked biological factors associated with natural immune responses to parasites also contribute to the higher incidence in males [11].

The epidemic of VL remains fundamentally uncontrolled in the region of this study, despite a basic control of the disease in China. This paper systematically explored the spatiotemporal characteristics and endemic trends from 2005 to 2017 using GIS and scan statistics advanced technology. Compared with surveys, both approaches have the advantages of availability and spatial continuity and, as ancillary analysis tools, have more extensive applicability in epidemiological studies when combined with local survey data. Public health policy-makers are generally interested in mapping the location of a disease and studying the disease spatial distribution in an area. The visualization of high-risk areas can guide managers to prioritize the optimal allocation of deployments, healthcare, and services [10]. The combination of epidemiological information and geospatial data allowed us to determine the locations of patients and spatial aggregations. Thus, in this study, all the methods that were used to assess the case location patterns confirmed that the distribution of VL cases was highly aggregated, highlighting the stability of the spatial distribution of the disease in the study area. ANN was used to explore the aggregation pattern of the cases, and the results provided the parameters for the analysis of the incremental spatial autocorrelation and hot-spot analysis. To make up for the ANN shortcomings, K-functions were adopted to investigate the clustering of the disease at different distances, and their calculated values can also serve as references for the hot-spot analysis. Incremental spatial autocorrelation was used to identify the best distance band that reflected maximum spatial autocorrelation for the hot-spot analysis. The maximum value of the *z*-score was 9.91, obtained for the distance of 2.75 km, which was very close to the radius of the B cluster. The Gi* statistic was used to recognize the VL hot spots based on the locations of the cases, and the tool of GIS provided a statistically stable and consistent method of detecting hot-spot and cold-spot areas in the region [12]. These results indicated that the VL cases showed local spatial aggregation, and the hot-spot area was in the central north of Jiashi County, whereas the cold spot areas were in the south-west of Jiashi County and the north-west of Yuepuhu County, thus confirming the focal occurrence of VL due to different local densities and the short-distance flight of phlebotomine vectors. Using this method and spatial visualization, the distribution of high-risk areas was clearly identified. 

The space–time scan statistics incorporated “time” to better comprehend the characteristics of the VL cases in the region, and the scan statistics identified three significant spatiotemporal clusters of VL cases with high incidence. Simple time clustering analysis only provides ambiguous information about whether clustering occurs at a certain time, and purely spatial analysis only provides spatial aggregation information without considering the time factor; however, spatiotemporal clustering analysis can not only detect the presence or absence of clustering but also simultaneously detect temporal and spatial aggregation. Spatiotemporal scan statistics compared with hot-spot analysis can be used to quantify the extent of a disease, the number of cases, and the relative risk in the scan window.

The spatiotemporal retrospective permutation model uses only case data without population-at-risk data and can analyze the spatiotemporal characteristics of an outbreak. The ability to perform disease surveillance without population-at-risk data is extremely significant in developing regions such as this study area in which these data may be difficult to obtain [13]. However, this method is highly sensitive to missing or incomplete data, and the geographic boundaries of detected outbreaks are not necessarily the same as those of true outbreaks [13]. In this study, because population-at-risk data were not obtained for each village, the attributable risk could not be calculated compared with that of the population, and relative risks were not calculated by the statistical test; therefore, these important clusters may or may not have a high relative risk. Additionally, the Oliveir-F metric could not be calculated because of the use of spatiotemporal permutation analysis to detect the cluster [14].

The temporal and spatiotemporal analysis provides a foundation to pursue future investigations of environmental factors. The results of hot-spot detection and spatiotemporal clustering patterns might offer some useful information to epidemiologists for the control of and to predict VL spread over critical hot-spot areas rather than in an entire region. Multiple causes are possible for the outbreaks of VL, and many previous studies have demonstrated that VL is highly sensitive to environmental factors and that climate affects the spatial and temporal distribution of the disease [15]. For instance, environmental factors, including forest cover, soil type, soil moisture, deforestation, and climate determinants, all play important roles in affecting the distribution of VL transmission [16,17].

**Figure 6:**
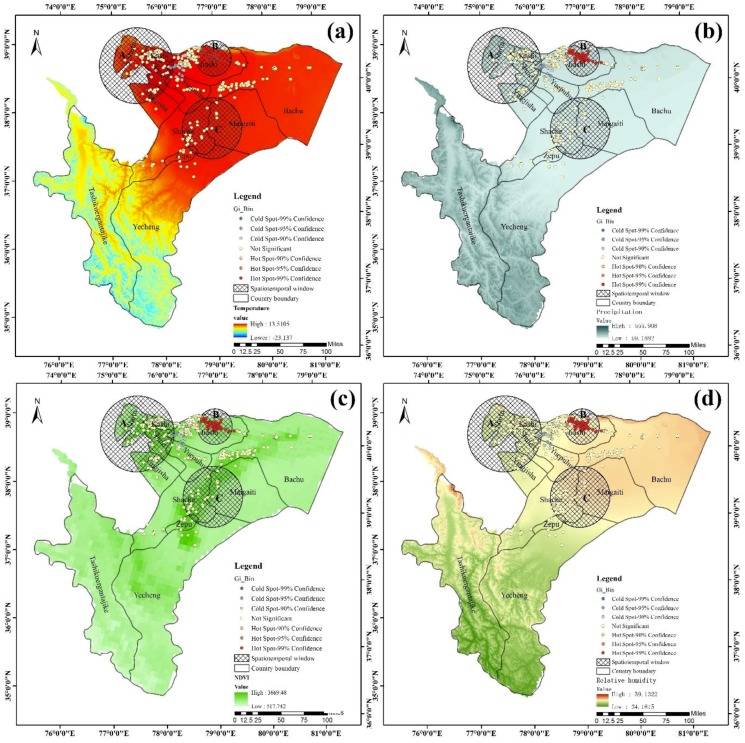
Mapping showing the relationships between VL cases’ distribution and environmental factors. Note: (**a**) represents the annual average temperature, (**b**) represents the annual average precipitation, (**c**) represents the annual average normalized difference vegetation index (NDVI), and (**d**) represents the annual average relative humidity from 2005 to 2017.

As indicated in Figure 6, the VL spatial cluster was distributed in areas in which the annual average temperature is above 10 degrees, and more VL cases occurred in areas with high temperatures than in areas with low temperatures. The rainfall range was from 91.996 to 105.176 mm, and more VL cases occurred in areas with low precipitation than in areas with high values. VL was primarily distributed in areas with a high normalized difference vegetation index (NDVI) compared with areas with a low value, indicating that vegetation might affect the distribution of the vehicle of the disease and therefore the transmission of VL. The relative humidity (RH) ranged from 36.82 to 48.69, and VL was primarily distributed in areas with high RH compared with those with low RH. However, in this study, the relationship between VL distribution and environmental factors in hot spots and spatiotemporal aggregation areas and the contribution of each environmental factor on it were not fully researched. Thus, further study should concentrate on investigating the relationships between geographic environmental factors and the spatiotemporal clusters of VL.

Additionally, some limitations of the research likely affected the results. First, the current surveillance system of VL in Kashi is not complete, and many patients are not diagnosed in health centers or reported to the surveillance system, leading to the possibility that a considerable proportion of VL cases was missed. The obtained official reports might underestimate the disease because VL was not reported, was not diagnosed, or was misdiagnosed [12]. Second, the population infected with VL without clinical symptoms is difficult to confirm, and that portion of the population might significantly affect the potential transmission of the disease, which might influence the spatial distribution of VL; however, in this study, this aspect was not considered [17]. Finally, it is well known that the pattern of VL cases are greatly influenced by the distribution of the sand fly, which is one of the primary modes of spread of VL and is widely spread in this region; therefore, the distribution pattern of the fly might affect the spatial distribution of VL. Meanwhile, the study conducted by Fu has proved that the seasonal dynamics of the anthroponotic VL is well correlated with the seasonal variation of *Phlebotomus* [18]. Therefore, the distribution pattern and characteristics of sand fly will be studied in future work.

## 5. Conclusions

The findings of this study indicate that VL continues to be a serious public health problem in Kashi Prefecture, China, particularly in the north-central region of Jiashi County, which is a relatively high-risk area in which hot spots are distributed. The results indicated that spatiotemporal clusters occurred in this region. Autumn and winter months were the outbreak season for VL cases. These findings provide a foundation to explore the relationship between the geographic environmental factors and the clusters of VL. Further study should concentrate on investigating the relationships between geographic environmental factors and the spatiotemporal cluster patterns of VL. The detection of spatiotemporal patterns can provide epidemiologists and local governments with significant information for prevention measures and control strategies. Furthermore, GIS and spatiotemporal permutation scan statistics to explore spatiotemporal characteristics and endemic trends can also be used for other similar endemic areas and are worth promoting. 

## Supplementary Materials

The following are available online at http://www.mdpi.com/1660-4601/15/12/2784/s1, Figure S1: Geographical distribution of all VL cases from 2005 to 2017, Document S1: Methodology of the study.

## Abbreviations

VL	visceral leishmaniasis
ANN	nearest-neighbour distance
GIS	Geographic Information System
WHO	World Health Organization
NDRIS	National Diseases Reporting Information System
