# Supplementary material for: Spatiotemporal Variation and Hot Spot Detection of Visceral Leishmaniasis Disease in Kashi Prefecture, China
Source: Int J Environ Res Public Health. 2018 Dec 8;15(12):2784. doi: 10.3390/ijerph15122784 (PMC6313707; doi:10.3390/ijerph15122784)
Supplement: Supplementary file 1 [file ijerph-15-02784-s001.zip › Supplement_Methodology.docx]

Methodology

Spatial statistical methods

Tobler's first law of geography states ‘Everything is related to everything else, but near things are more related than distant things' [1]. This dependence rule is often called spatial autocorrelation. In the case of spatial autocorrelation, the null hypothesis states that ‘there is no spatial pattern among the VL cases’, and to evaluate whether a pattern is clustered, dispersed or random, different tests have been proposed [2]. For this purpose, five different methods, namely, the Average Nearest Neighbour distance (ANN), Moran's I, Ripley's K‐function, the Incremental Spatial Autocorrelation and the Getis-Ord Gi* statistic, were used to detect the clustering of VL; for the detailed description, please refer to the attachment.

Global Moran's I

Moran's I Index statistic was used for the measurement of spatial autocorrelation [3]. Significance of the index is assessed using both the z-score and P-value. The values of Moran’s I range from −1 to +1, and Moran’s I > 0, = 0, and < 0 indicate positive spatial autocorrelation, random distribution, and negative spatial autocorrelation, respectively [4]. The z-score was used to decide whether to reject the null hypothesis, and the probability of a false rejection was tested by the p-value [5]. Moran's I has been widely used in epidemiology, including in studies on haemorrhagic fever [6], leishmaniasis [7], human brucellosis [8], and the under-five mortality rate [9]. Moran's I adopts a covariance term between each point and its neighbours as follows (Mitchell, 2005):

 (1)

 (2)

Where n is the total number of cases; w_i,j_ is the spatial weight between the cases i and j; x_i_ and x_j_ are the numbers of VL cases in the i^th^ and j^th^ points, respectively; and w_ij_ is the spatial neighbourhood weight for points i and j. The weight is defined based on adjacent neighbours as shown in the following equation [5]:

$\text{w}_{\text{ij}}=\left\{\begin{matrix} 1 \\ 0 \end{matrix}\right.$ (3)

otherwise

If i, j are adjacent neighbours

Afterwards, the weight matrix is standardized by row, i.e., every neighbour weight for a point is divided by the sum of all neighbour weights.

Average nearest neighbour

The ANN measures the distance between each feature centroid and its nearest neighbour's centroid location. All these nearest neighbour distances are then averaged. If the average distance is less than the average for a hypothetical random distribution, the distribution of the features being analysed is considered clustered. If the average distance is greater than a hypothetical random distribution, the features are considered dispersed [10]. The average nearest neighbour ratio is calculated as the observed average distance divided by the expected average distance. The results of the calculation can be used as parameters for the incremental autocorrelation spatial and hot spot analyses.

Ripley's K‐function

The ANN measure has a major drawback because it only makes use of the nearest neighbour for each case in a pattern for clustered patterns in which nearest‐neighbour distances are very short relative to other distances in the pattern [11]. Ripley's K-function is an efficient method that analyses multidistance spatial clusters and was used to measure global spatial autocorrelation with the original point data [12] A. Mollalo et al. applied the K‐function method to investigate the annual spatial distribution of the existing ZCL case point patterns [7], and Sarah E Hinman applied Ripley's K-function to evaluate spatial clustering [12].

Hotspot detection and analysis

Global indices do not specify the location of cluster(s). To test for statistically significant local VL clusters and to determine the general spatial extent of those clusters, we used the Getis-Ord Gi* statistical tool [13,14]. In this study, the Integrate and Collect Events method was used to integrate points of VL cases, and then the incremental spatial autocorrelation tool was used to help identify a distance band that reflected maximum spatial autocorrelation as the scale for hot spot analysis [15]. The Getis-Ord Gi* statistic was used to identify VL clusters of high values from clusters of low values. Moreover, clusters of cases that occur randomly can also have an influence on the spread of an infectious disease [4]. The Gi * statistic is written as follows [15]：

$Gi*=\frac{\sum_{j=1}^{n} w_{i,j}x_{j}-\overline{X}\sum_{j=1}^{n} w_{i,j}}{S\sqrt{\frac{n\sum_{j=1}^{n} w_{i,j}^{2}-\left(\sum_{j=1}^{n} w_{i,j}\right)}{n-1}}}$ (4)

$\overline{X}=\frac{\sum_{\text{j}=1}^{\text{n}} \text{x}_{\text{j}}}{\text{n}}$ (5)

$S=\sqrt{\frac{\sum_{\text{j}=1}^{\text{n}} \text{x}_{\text{j}}^{2}}{\text{n}}}\text{-}(\overline{X})^{2}$ (6)

where x_j_ is the number of VL cases in the area j, w_i_, j is the spatial weight between points i and j, and n is the total number of points.

The Gi* statistic is a z-score, and therefore, no further calculations are required. The output from the Gi * statistic identifies spatial clusters of high values (hot spots) and spatial clusters of low values (cold spots) and provides confidence level bins (Gi_Bin) with features in the +/-3; +/-2; and +/-1 bins statistically significant at the 99%, 95%, and 90% confidence levels, respectively. Spatial aggregation for features with 0 for the Gi_Bin field was not statistically significant [16].

Spatiotemporal permutation scan statistics

In this research, the spatiotemporal permutation scan statistic was used in the SaTScan software version 9.5, which is freely available from [www.satscan.org](http://www.satscan.org) [17]. The spatiotemporal permutation model introduced by Kulldorff was applied to analyse a space-time featured variable [18]. This model does not require population-at-risk data and can be used for the early detection of disease outbreaks when only the number of cases is available. Scan statistics are used in a retrospective way to detect past clusters using retrospective data and in a prospective way to detect clusters at the present time [17]. Scan statistics are explained by a cylindrical window with a circular geographical basis and the height indicating time. The window moves in space and time and therefore covers each potential time span for each geographical location resulting in defining an infinite number of overlapping cylinders of different forms and sizes that finally cover the entire study area.

The Poisson generalized likelihood ratio was used to estimate the likelihood of a cluster in a given spatiotemporal cylinder. Finally, Monte Carlo permutation was used to test for the significance level of clusters. In this analysis, values for the geographical size are 50% of the total population-at-risk and for the spatial size, 50% of the total time period [19]. Scan statistics were based on the monthly VL date of onset. Spatiotemporal retrospective analysis was used, and the circular spatial window shape was set to standard, and the coordinate system was latitude/longitude. The number of replications was 9999. Clusters with high incidence rates were detected, and only clusters with no geographical overlap were reported.

Reference

1. A Computer Movie Simulating Urban Growth in the Detroit Region; 1970.

2. Mitchel A. E. The ESRI Guide to GIS analysis, Volume 2: Spartial measurements and statistics. *Esri Guide to Gis Analysis* 2005

3. Anselin L, Getis A. Spatial statistical analysis and geographic information systems. *Annals of Regional Science* 1992;26(1):19-33.

4. Bhunia GS, Kesari S, Chatterjee N, et al. Spatial and temporal variation and hotspot detection of kala-azar disease in Vaishali district (Bihar), India. *Bmc Infectious Diseases* 2013;13(1):64.

5. Ahmadkhani M, Alesheikh AA, Khakifirouz S, et al. Space-time epidemiology of Crimean-Congo hemorrhagic fever (CCHF) in Iran. *Ticks Tick Borne Dis* 2017;9(2)

6. Abbas T, Younus M, Muhammad SA. Spatial cluster analysis of human cases of Crimean Congo hemorrhagic fever reported in Pakistan. *Infect Dis Poverty* 2015;4(1):9.

7. Mollalo A, Alimohammadi A, Shirzadi MR, et al. Geographic information system-based analysis of the spatial and spatio-temporal distribution of zoonotic cutaneous leishmaniasis in Golestan Province, north-east of Iran. *Zoonoses & Public Health* 2015;62(1):18-28.

8. Mollalo A, Alimohammadi A, Khoshabi M. Spatial and spatio-temporal analysis of human brucellosis in Iran. *Trans R Soc Trop Med Hyg* 2014;108(11):721-28.

9. Li Z, Fu J, Jiang D, et al. Spatiotemporal Distribution of U5MR and Their Relationship with Geographic and Socioeconomic Factors in China. *International Journal of Environmental Research & Public Health* 2017;14(11):1428.

10. ESRI. Average Nearest Neighbor (Spatial Statistics)—Help | ArcGIS for Desktop [Internet] ArcGis for desktop2018 [cited 2018 May]. Available from: ttp://resources.arcgis.com/en/help/main/10.2/index.html#/na/005p00000008000000/ accessed Consuilted May 2018.

11. O'sullivan D, Unwin D. Geographic information analysis: John Wiley & Sons 2014.

12. Hinman SE, Blackburn JK, Curtis A. Spatial and temporal structure of typhoid outbreaks in Washington, DC, 1906–1909: evaluating local clustering with the G i* statistic. *International Journal of Health Geographics* 2006;5(1):13.

13. Ord JK, Getis A. Local Spatial Autocorrelation Statistics: Distributional Issues and an Application. *Geographical Analysis* 1995;27(4):286-306.

14. Ma LG, Chen QH, Wang YY, et al. Spatial pattern and variations in the prevalence of congenital heart disease in children aged 4-18 years in the Qinghai-Tibetan Plateau. *Science of the Total Environment* 2018;627:158-65.

15. ESRI. Hot Spot Analysis (Getis-Ord Gi*)—Help | ArcGIS for Desktop [Internet] ArcGis for desktop2018 [updated Consulted May 2018. Available from: <http://resources.arcgis.com/zh-cn/help/main/10.2/index.html#/na/005p00000011000000/> accessed Consulted May 2018.

16. Blanco-Guillot F, Castañeda-Cediel ML, Cruz-Hervert P, et al. Genotyping and spatial analysis of pulmonary tuberculosis and diabetes cases in the state of Veracruz, Mexico. *PloS one* 2018;13(3):e0193911.

17. Kulldorff M, Heffernan R, Hartman J, et al. A space–time permutation scan statistic for disease outbreak detection. *PLoS medicine* 2005;2(3):e59.

18. Kulldorff M. SaTScan-Software for the spatial, temporal, and space-time scan statistics. *Boston: Harvard Medical School and Harvard Pilgrim Health Care* 2010

19. Zare M, Rezaianzadeh A, Tabatabaee H, et al. Spatiotemporal clustering of cutaneous leishmaniasis in Fars province, Iran. *Asian Pacific Journal of Tropical Biomedicine* 2017;7(10):862-69.
