# Supplementary figures and images for: Spatiotemporal Variation and Hot Spot Detection of Visceral Leishmaniasis Disease in Kashi Prefecture, China
Source: Int J Environ Res Public Health. 2018 Dec 8;15(12):2784. doi: 10.3390/ijerph15122784 (PMC6313707; doi:10.3390/ijerph15122784)

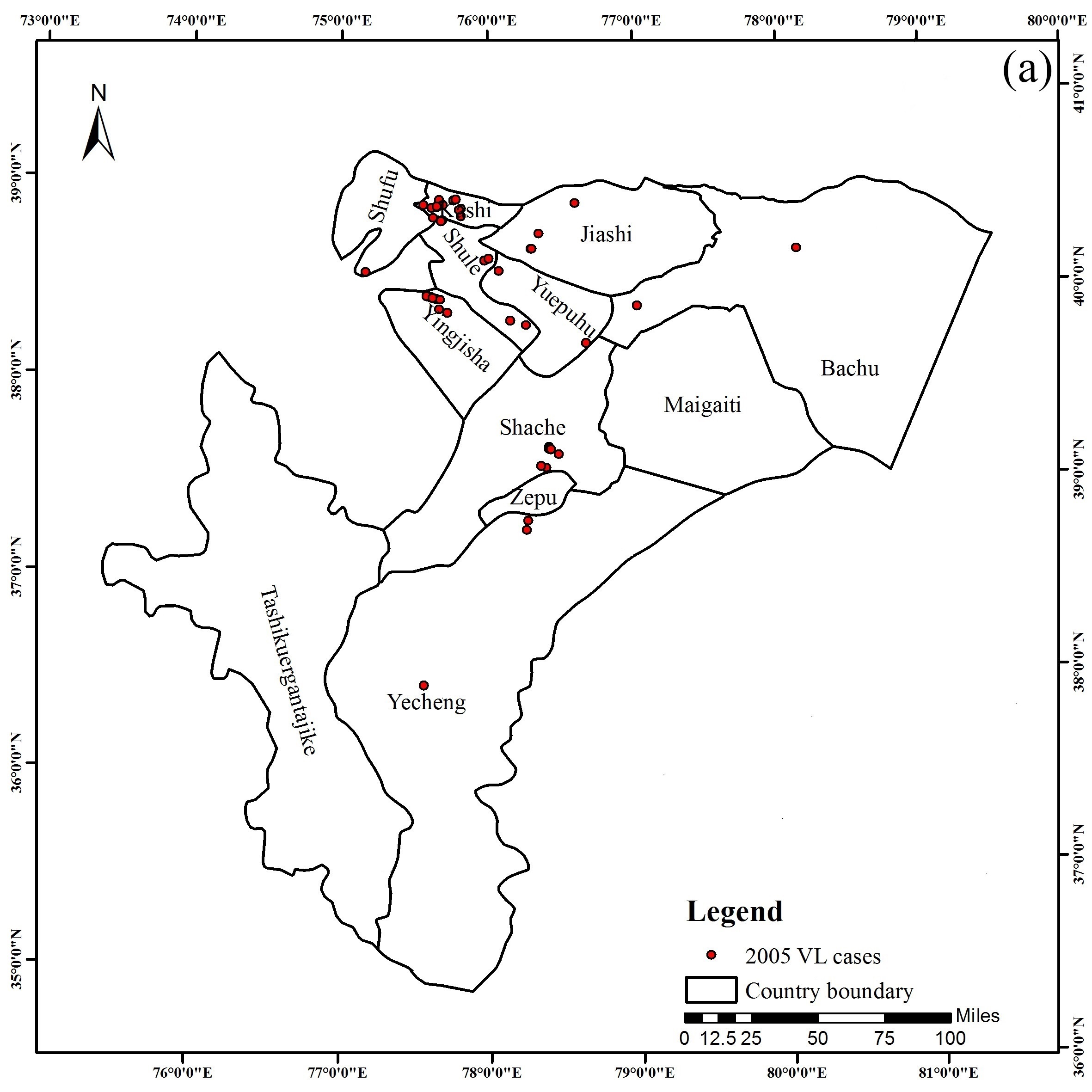

Supplement: Supplementary file 1 [file ijerph-15-02784-s001.zip › Supplement Figure (a).jpg]

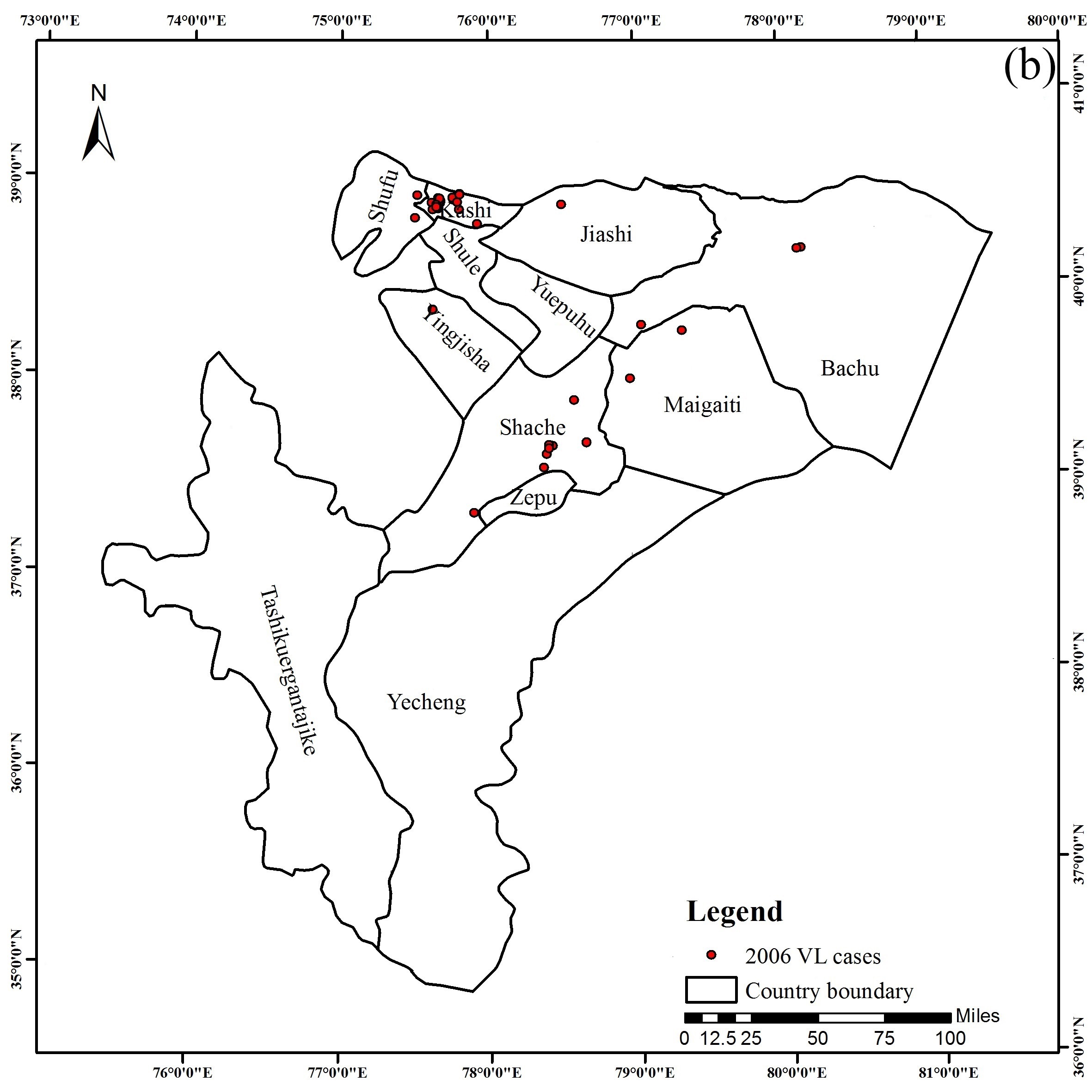

Supplement: Supplementary file 1 [file ijerph-15-02784-s001.zip › Supplement Figure (b).jpg]

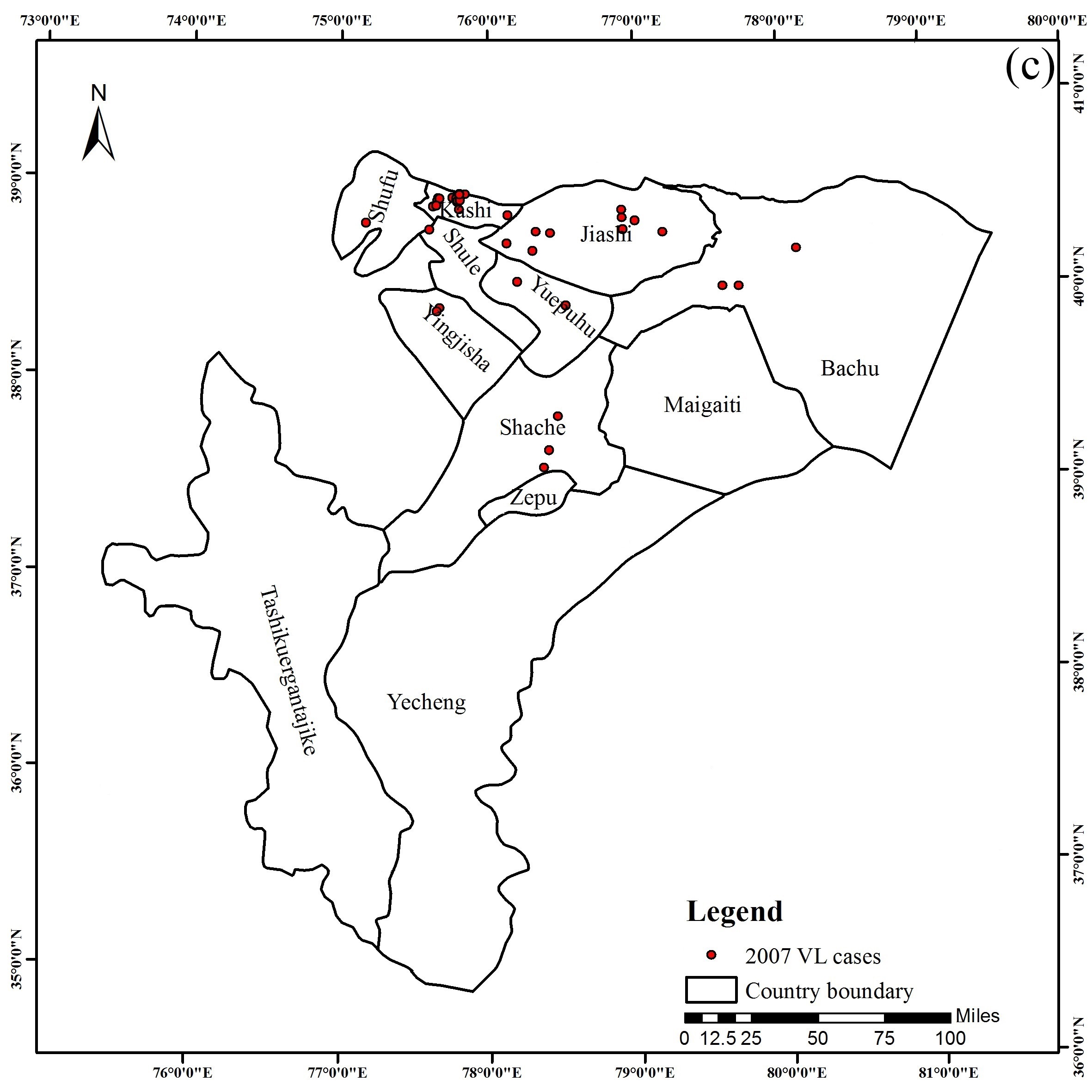

Supplement: Supplementary file 1 [file ijerph-15-02784-s001.zip › Supplement Figure (c).jpg]

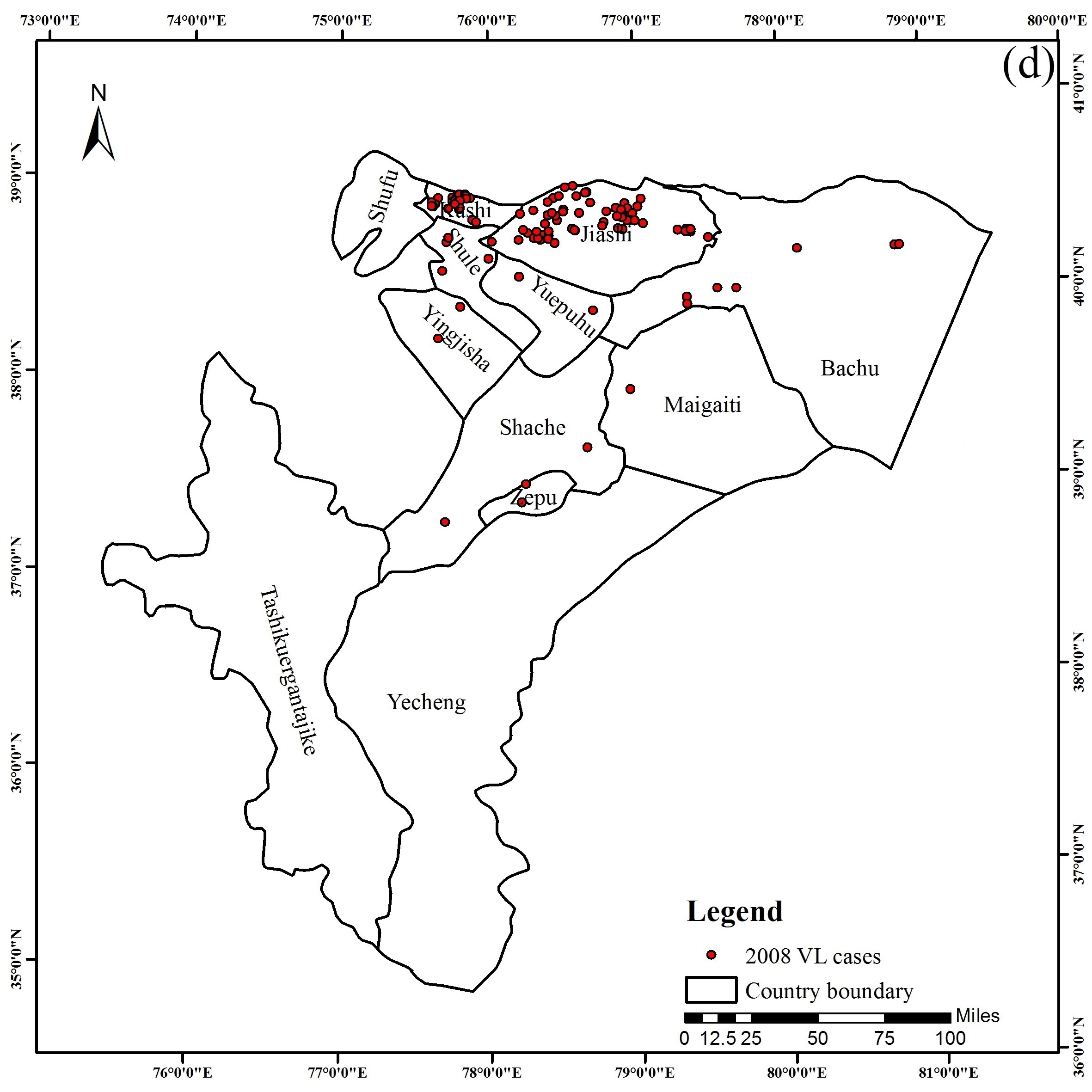

Supplement: Supplementary file 1 [file ijerph-15-02784-s001.zip › Supplement Figure (d).jpg]

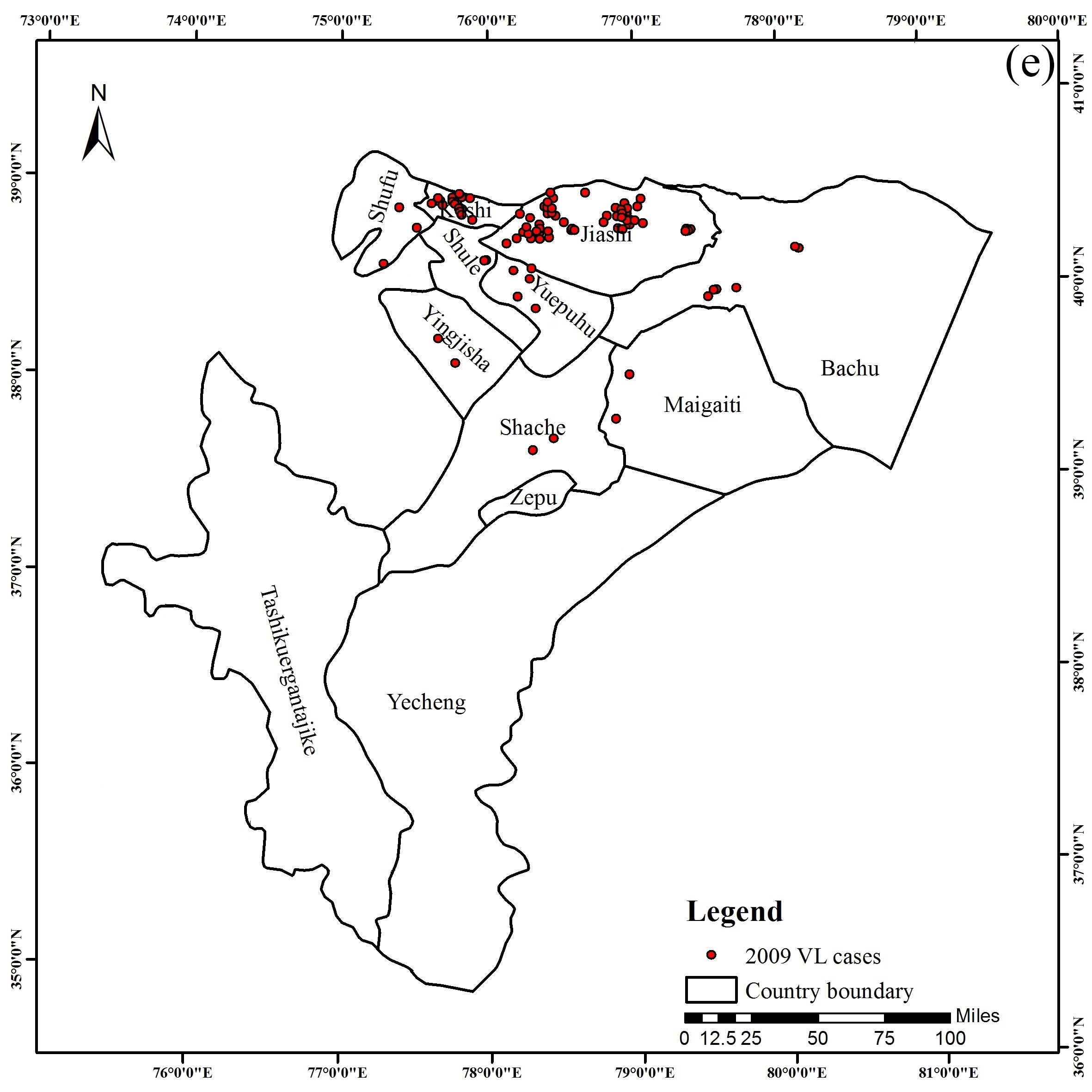

Supplement: Supplementary file 1 [file ijerph-15-02784-s001.zip › Supplement Figure (e).jpg]

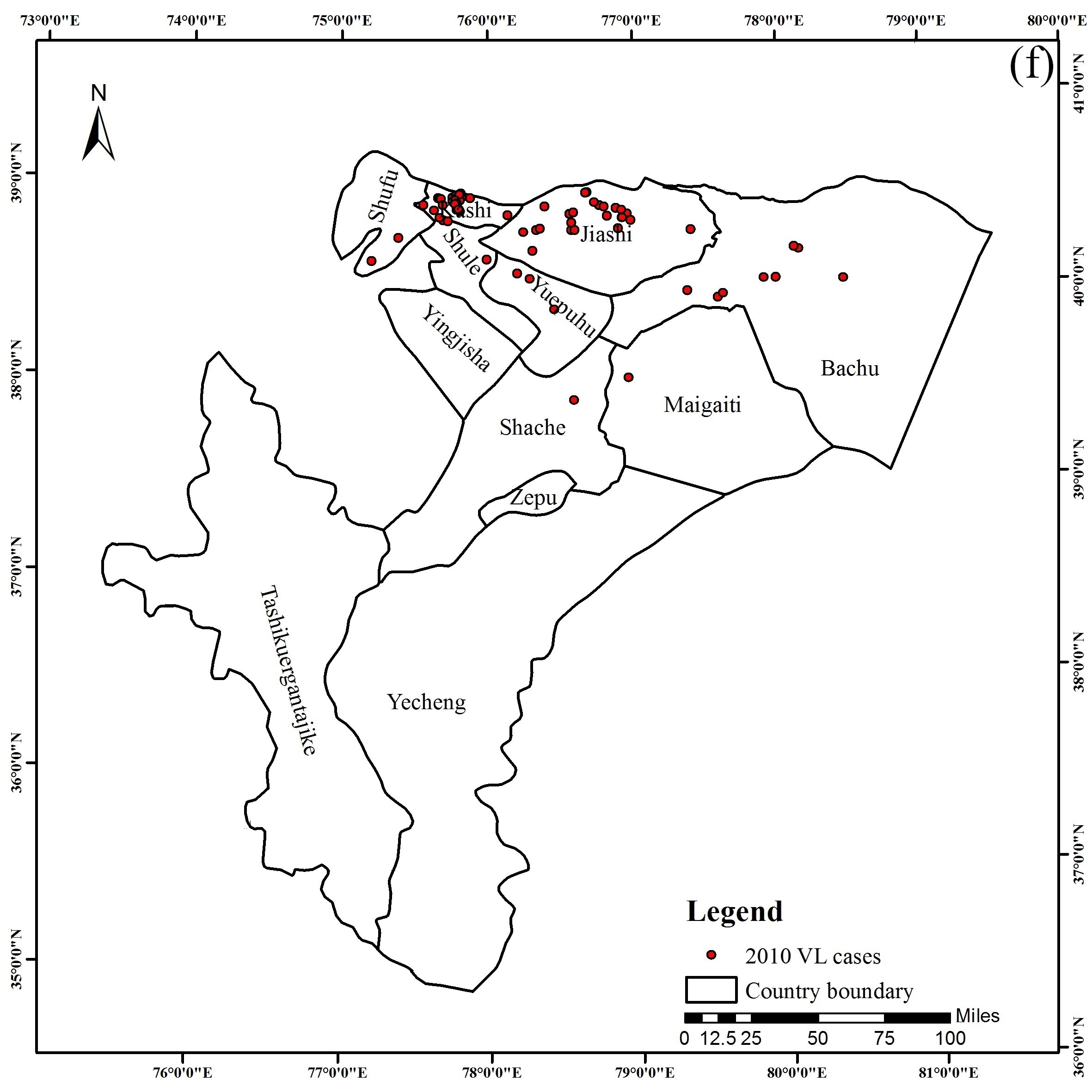

Supplement: Supplementary file 1 [file ijerph-15-02784-s001.zip › Supplement Figure (f).jpg]

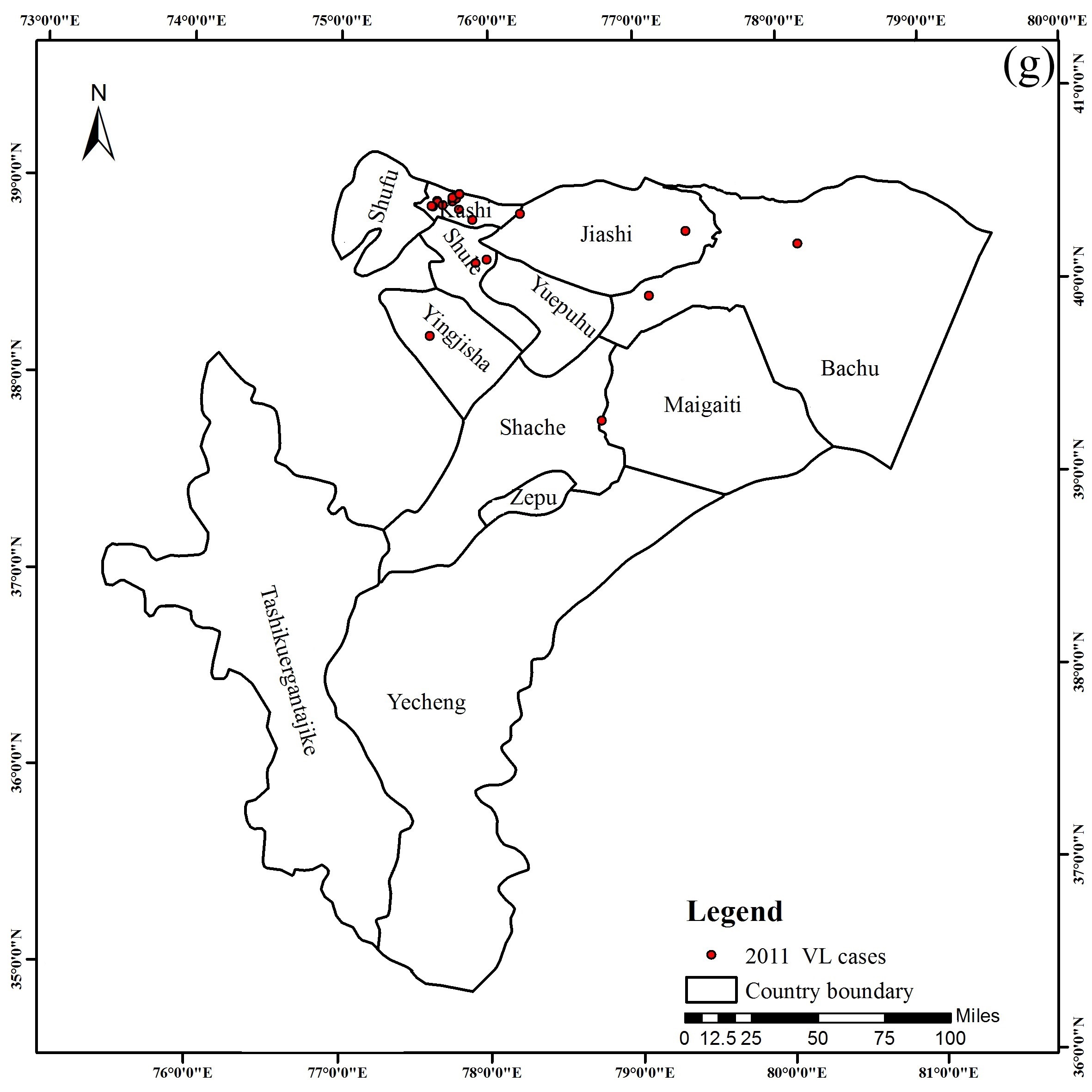

Supplement: Supplementary file 1 [file ijerph-15-02784-s001.zip › Supplement Figure (g).jpg]

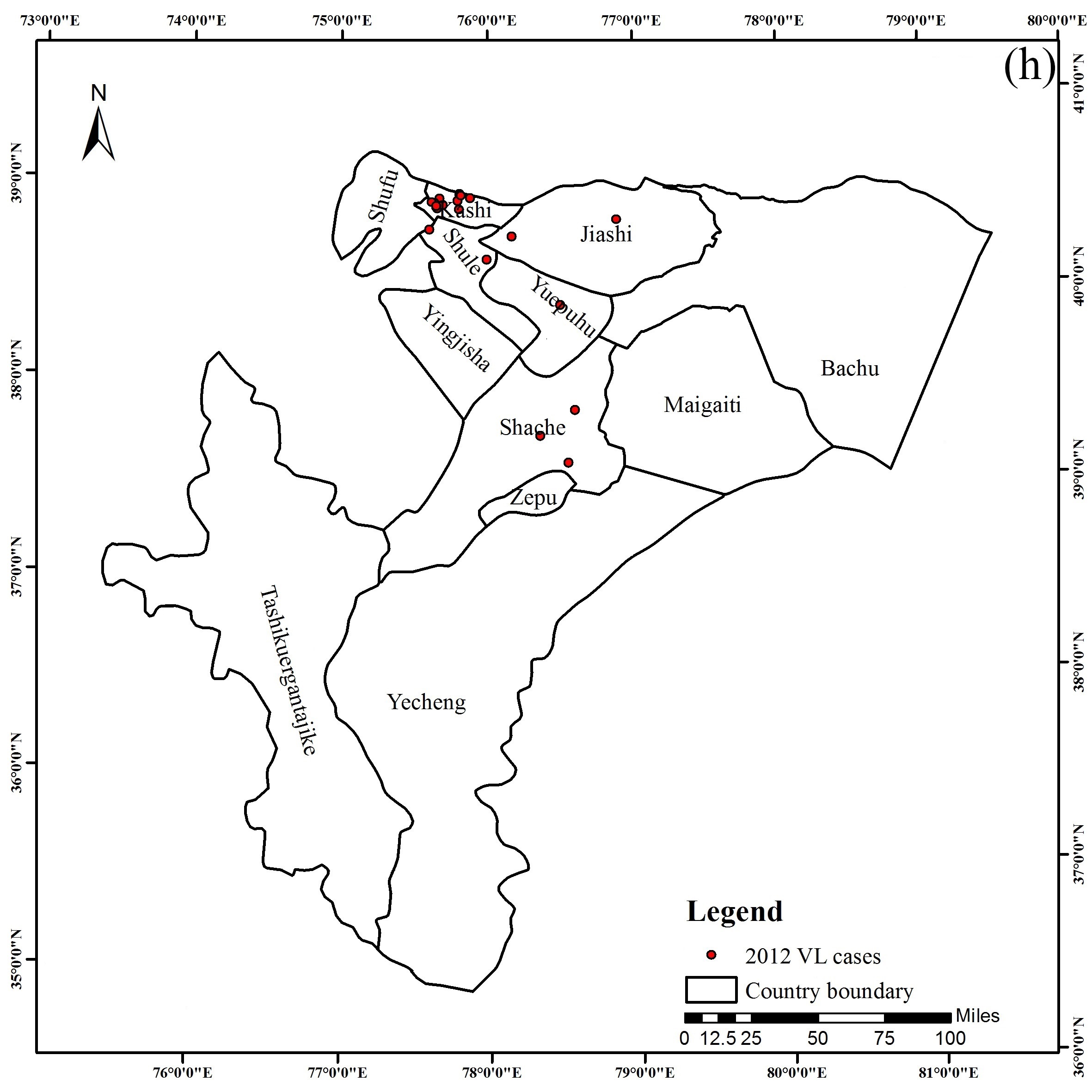

Supplement: Supplementary file 1 [file ijerph-15-02784-s001.zip › Supplement Figure (h).jpg]

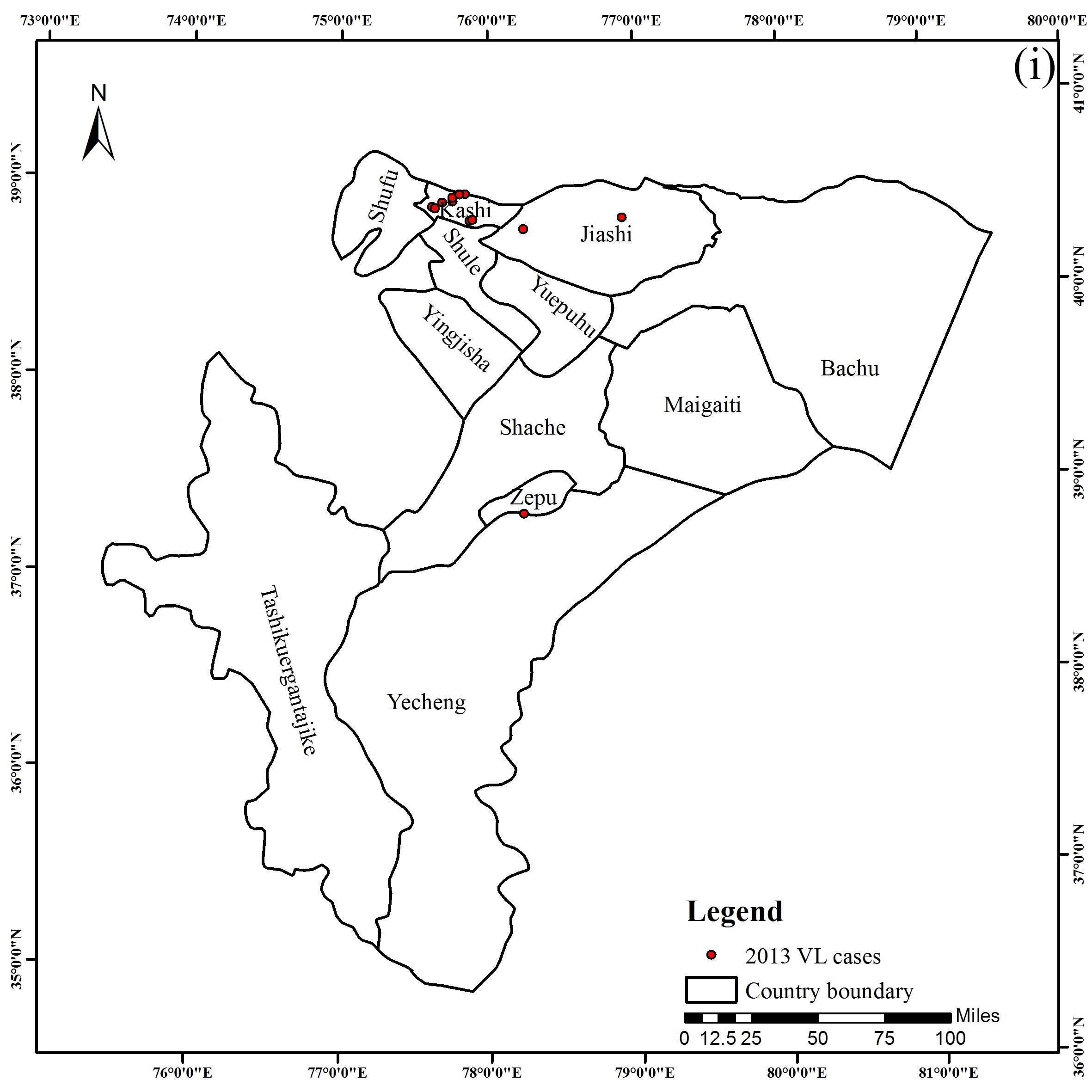

Supplement: Supplementary file 1 [file ijerph-15-02784-s001.zip › Supplement Figure (i).jpg]

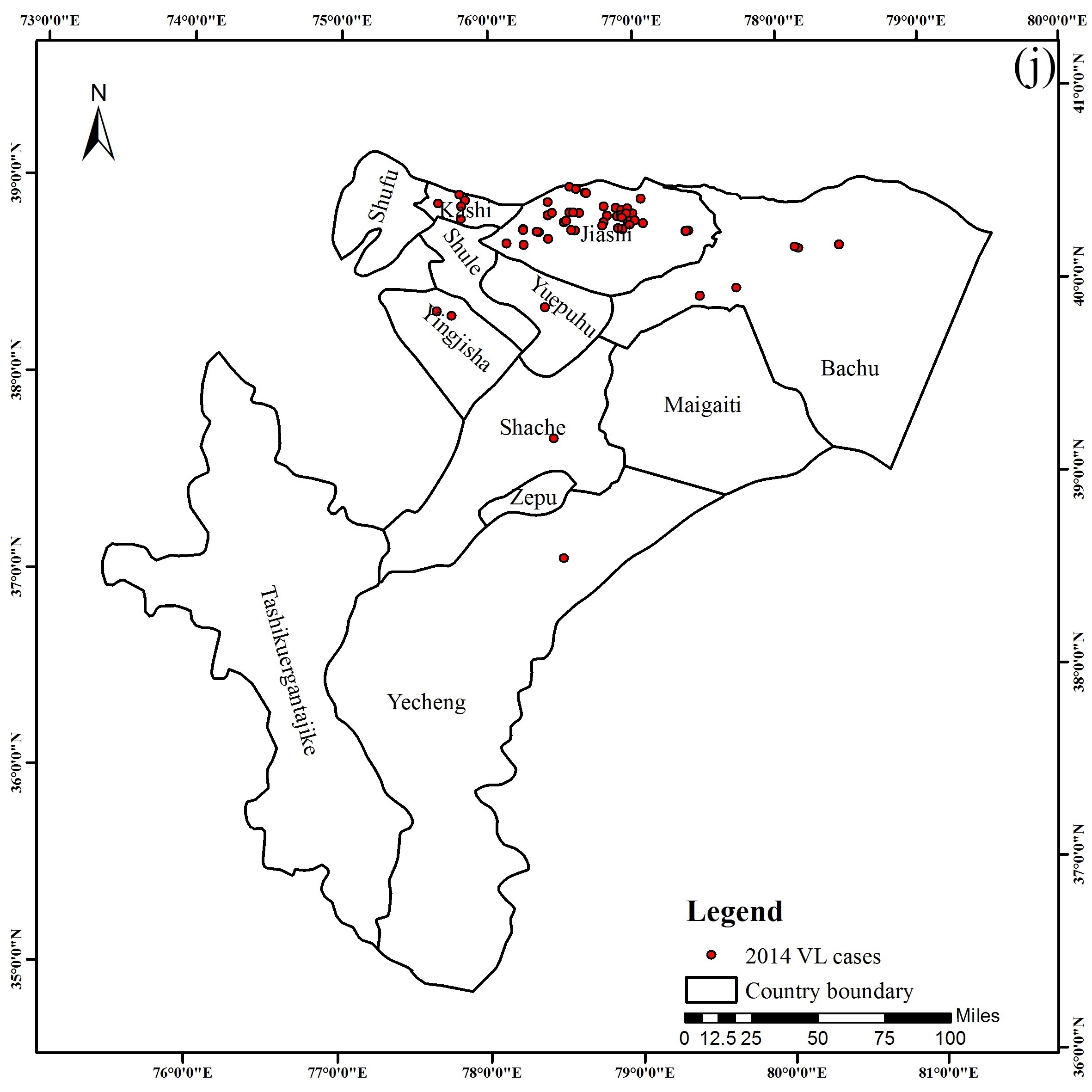

Supplement: Supplementary file 1 [file ijerph-15-02784-s001.zip › Supplement Figure (j).jpg]

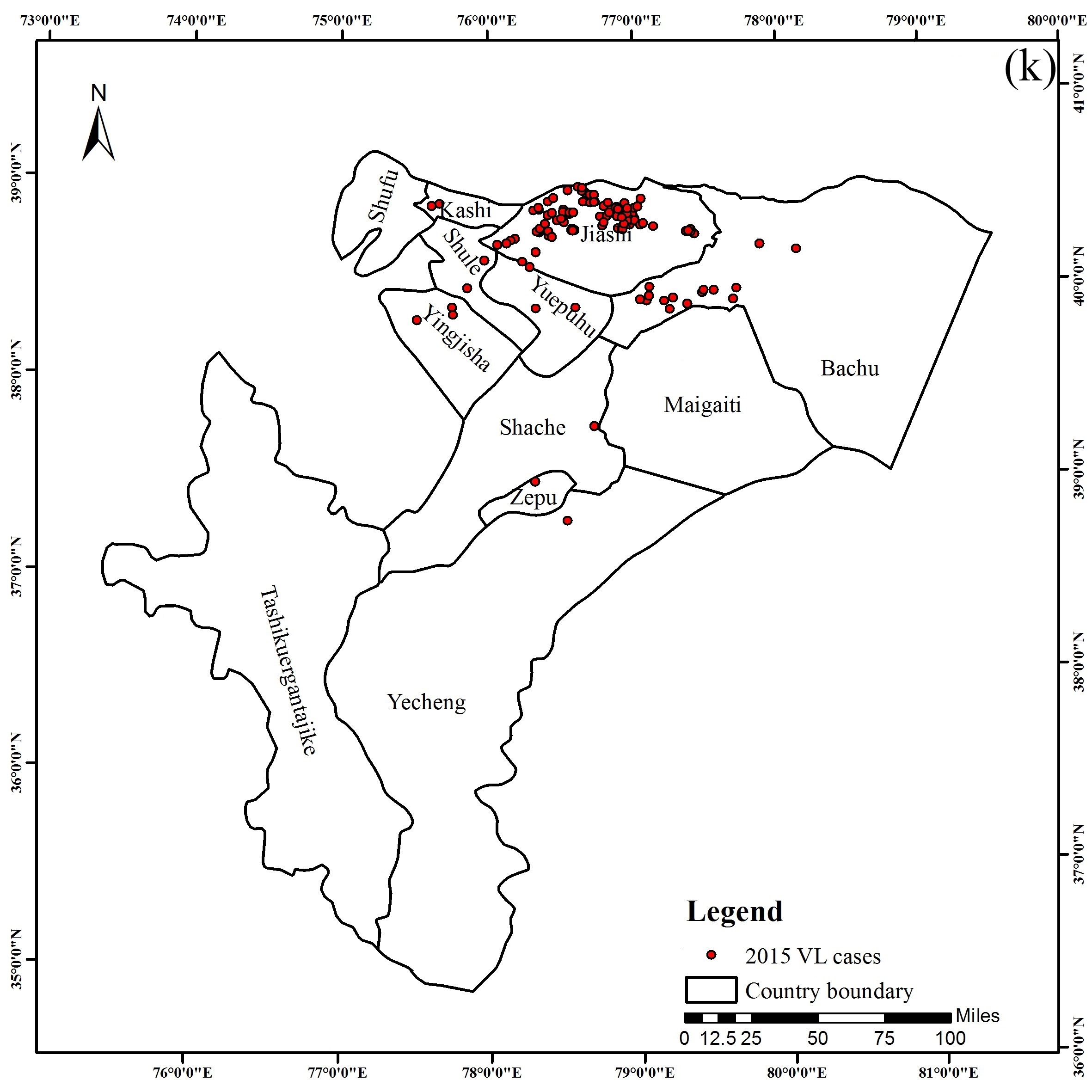

Supplement: Supplementary file 1 [file ijerph-15-02784-s001.zip › Supplement Figure (k).jpg]

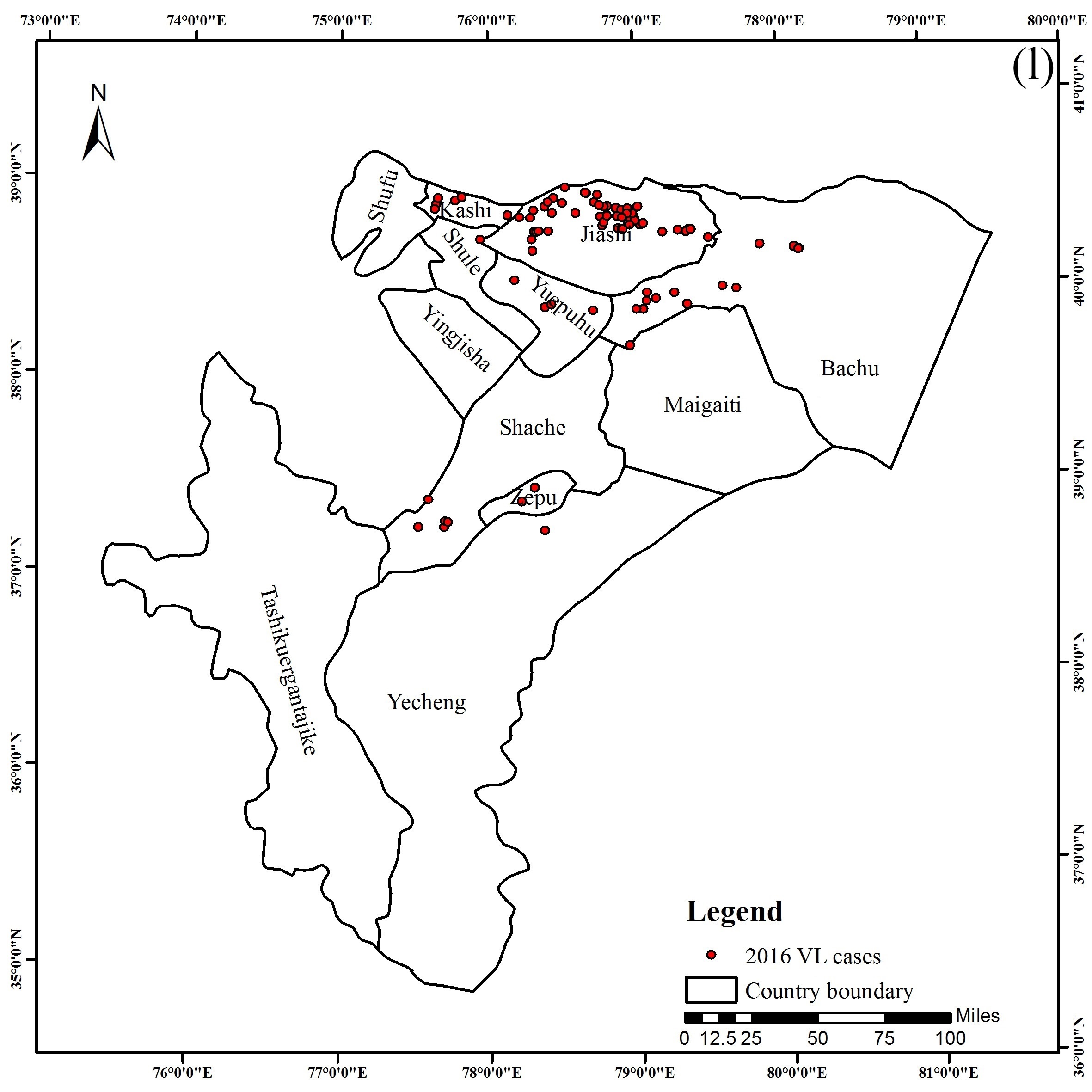

Supplement: Supplementary file 1 [file ijerph-15-02784-s001.zip › Supplement Figure (l).jpg]

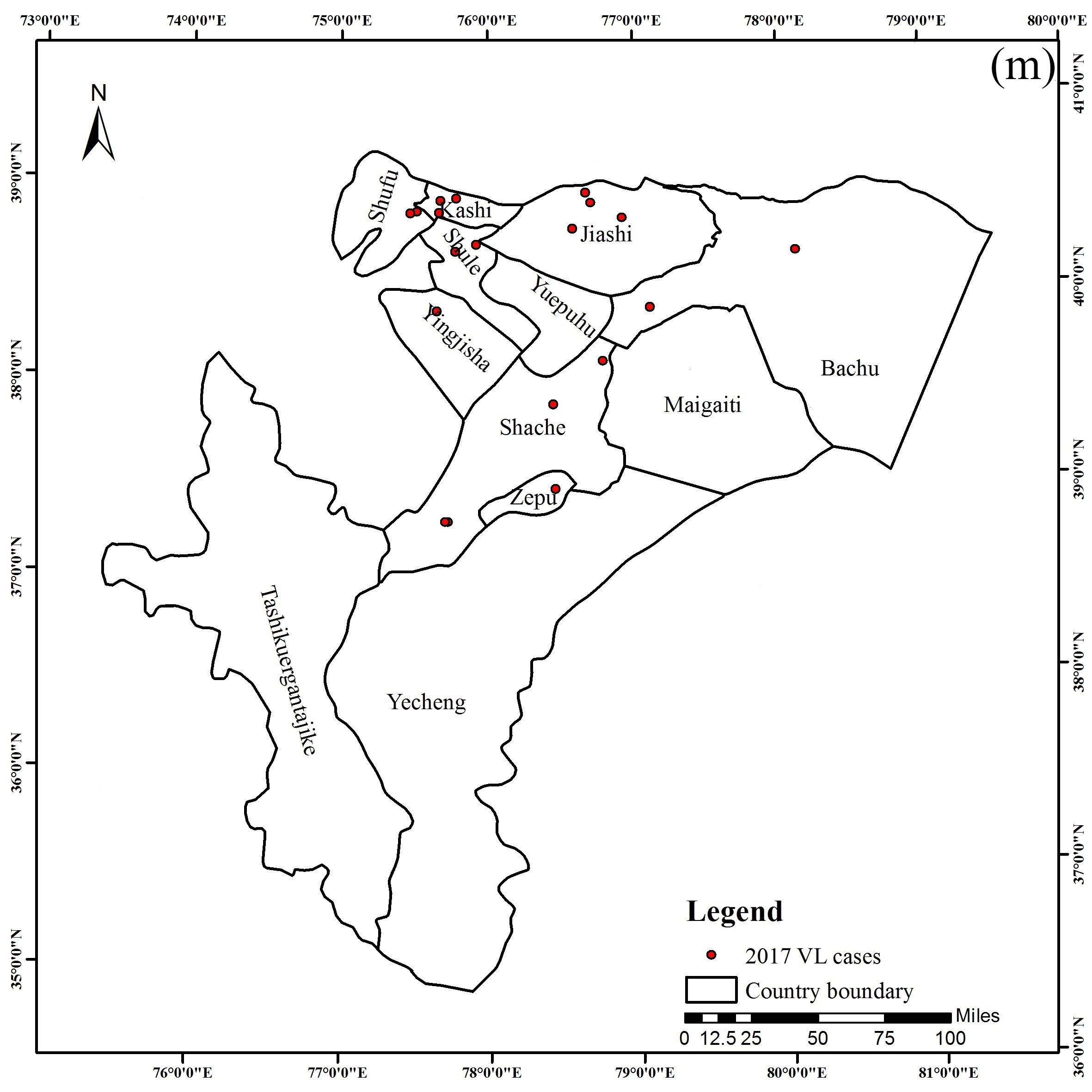

Supplement: Supplementary file 1 [file ijerph-15-02784-s001.zip › Supplement Figure (m).jpg]
